# *In-silico* Metabolome Target Analysis Towards *PanC*-based Antimycobacterial Agent Discovery 

**Published:** 2015

**Authors:** Baharak Khoshkholgh-Sima, Soroush Sardari, Jalal Izadi Mobarakeh, Ramezan Ali Khavari-Nejad

**Affiliations:** a *Department of Biology, Science and Research Branch, Islamic Azad University, Tehran, Iran. *; b *Drug Design and Bioinformatics Unit, Medical Biotechnology Department, Biotechnology Research Center, Pasteur Institute of Iran Tehran, Iran. *; c *Department of Pharmacology, Tehran Medical Science Branch, Islamic Azad University, Tehran, Iran.*

**Keywords:** *Mycobacterium*, Metabolic pathway, Pantothenate synthetase (*PanC*), Antimycobacterial agents

## Abstract

*Mycobacterium tuberculosis*, the main cause of tuberculosis (TB), has still remained a global health crisis especially in developing countries. Tuberculosis treatment is a laborious and lengthy process with high risk of noncompliance, cytotoxicity adverse events and drug resistance in patient. Recently, there has been an alarming rise of drug resistant in TB. In this regard, it is an unmet need to develop novel antitubercular medicines that target new or more effective biochemical pathways to prevent drug resistant *Mycobacterium*.

Integrated study of metabolic pathways through *in-silico* approach played a key role in antimycobacterial design process in this study. Our results suggest that pantothenate synthetase (*PanC*), anthranilate phosphoribosyl transferase (*TrpD*) and 3-isopropylmalate dehydratase (*LeuD*) might be appropriate drug targets. In the next step, *in-silico* ligand analysis was used for more detailed study of chemical tractability of targets. This was helpful to identify pantothenate synthetase (*PanC, Rv3602c*) as the best target for antimycobacterial design procedure. Virtual library screening on the best ligand of *PanC* was then performed for inhibitory ligand design. At the end, five chemical intermediates showed significant inhibition of *Mycobacterium bovis* with good selectivity indices (SI) ≥10 according to Tuberculosis Antimicrobial Acquisition & Coordinating Facility of US criteria for antimycobacterial screening programs.

## Introduction

Tuberculosis has remained one of the major lethal infectious diseases which kill more people in comparison to any other microbial pathogen**. **This is happening while a broad range of effective drug regime is available worldwide (1). Current antituberculars have encountered numerous challenges such as drug resistance, HIV co-infection and persistence state. These drawbacks highlight the unmet needs for drug candidate which act through new biochemical pathways (1-3). Sirturo, which inhibits mycobacterial F1F0-adenosine triphosphate (ATP) synthase, is the first drug of new class of antitubercular drugs with novel mechanism that has been approved in the last 40 years. This drug specifically addresses *Mycobacterium* multi-drug resistance. However, the main challenge to produce this new antimycobacterial was the fact that, Sirturo increases risks of potentially fatal abnormal heart rhythms (4, 5). Considering problems mentioned above in tuberculosis treatment, proposing new drug candidates which address these critical issues is believed to be a health priority and has attracted attention of researchers. As a result, finding new pathways and development of novel drugs addressing them are rather crucial in tuberculosis management (3).

Information-processing enzymes such as DNA polymerase, RNA polymerase and DNA gyrase are the ones which were widely used in drug design. Metabolic pathways have acquired less attention in antituberculars in recent years (6). Therefore, metabolic pathways have remained as a fruitful avenue for the development of new antituberculars which may address existing drugs shortcomings.

This study focused on characterization of more promising target as a solid base for antimycobacterial design. *In-silico* analysis has been reported as more efficient method in comparison to time-consuming and expensive experimental investigations of possible drug targets. *In-silico* analysis of metabolome targets was employed through integration and application of datasets in different biological domains. This broad range of selection criteria covers different aspects of possible targets to better identify plausible drug candidates. *In-silico* ligand analysis was then applied to select most suitable targets for further cheminformatics follow up. This procedure was continued by a virtual screening approach in NIH PubChem Database for identification of virtual library hits. Ultimately, the hit molecules were prepared and assayed against Bacillus Calmette–Guérin (BCG) to obtain the antimycobacterial activity. Additionally, this approach was pursued through cytotoxicity assay in Human Umbilical Vein Endothelial Cell line.

## Experimental

*Materials*

Chemical intermediates were purchased from Merck Company (Germany) and Sigma Company (Germany). The Human Umbilical Vein Endothelial (HUVEC) cell line (NCBI Code: C554) and BCG (1173P2) were obtained from Pasteur Institute (Iran). RPMI 1640, streptomycin, penicillin and fetal bovine serum (FBS) were purchased from Gibco Invitrogen Company (USA). Trypan blue was provided from Merck Company (Germany). Ethambutol, alamar blue and tween 80, dimethyl sulfoxide (DMSO) 99.7% purchased from Sigma Company (Germany). Middlebrook 7H9 Broth Base was obtained from HiMedia (India). 

*Methods*

*Selection of drug target candidates via *integrated* criteria*

This target identification approach comprised integrating datasets from different biology aspects related to criteria such as known function, essentiality, choke points, non-homology and involvement in persistence and virulence. Selection of drug targets was then performed via integrated criteria, in which only targets of metabolic pathways which met all desired criteria were selected. 

Targets function was obtained through bioinformatic resources including KEGG (Kyoto Encyclopedia of Genes and Genomes) (7) and TubercuList (*Mycobacterium* Tuberculosis Database) (8). We discriminated between known and unknown functions for rational drug design and complementary future studies through biochemical or biophysical methods.

Essential genes were used in order to identify key genes in *Mycobacterium* growth and survival (9). Pervious experimental Datasets of transposon site hybridisation (TraSH) mutagenesis technique in H37Rv and CDC1551 were extracted from TubercuList database (8) to choose essential genes for *Mycobacterium* growth and survival.

Metabolic choke points, which uniquely consume or produce a particular metabolite, was used to identify potential targets based on the biochemical lethality of metabolic networks (10). Identification metabolic choke points reaction that non-compensated by alternative pathways was performed according to Kushwaha and Shakya experimental dataset (11).

Non-homology criterion is important to identify possible interference of drugs with the human genes which might lead to adverse side effects (9). Identification of non-homologous targets was performed according to Anishetty dataset (12). 

In addition, the increasing emergence of persistent bacteria highlights the need to develop novel TB bactericides that shorten treatment length (3). Unfortunately, most of current drugs are interesting only because of their activity against growing *Mycobacterium tuberculosis* (13). Therefore, persistence is still a critical criterion in selection of drug target candidates to obtain ability to combat with persistent bacteria. Expression during persistence was found through bioinformatics resource; include TB Database (14).

Virulence factors (VF) is another criterion to identify of drug target candidates. These targets play a key role in establishment and severity of infection, so that inhibition of these virulence factors would make the pathogen avirulent (15). Identification of virulence factors was done from Virulence Factor DataBase (VFDB) that comprises virulence factors of 24 species of pathogenic bacteria, include *Mycobacterium* genus (15, 16).

*In-silico ligand analysis*

Ligands of selected drug targets were considered to obtain promising ones for follow up. Therefore, two distinct parameters of enzyme activity and Lipinski rule of five were used to evaluate the ligand. Enzyme activity was used to assay acceptable potencies and associated ligands efficiencies through their activity against enzyme. Physicochemical parameters were also assayed based on Lipinski rule of five as a method of quantifying drug likeness. According to Lipinski rule of five, only ligands that violate just one role may be regarded as drug like ligands. These rules include (i) no more than five H-bond donors, (ii) no more than 10 H-bond acceptors, (iii) molecular weight no higher than 500 Daltons, and (iv) calculated primary predictive index of lipophilicity (XlogP) no more than five (17). 

*Ligand-based virtual screening*

Virtual screening has become attractive in computational filtering of comprehensive databases like Pubchem in order to evaluate compounds properties, identify preferred compounds and eliminate those having undesired features (18).

In this study, ligand-based virtual screening protocol was applied using ligand of the selected target candidate. Filtration strategy comprised structural similarity, Lipinski rule of five and mycobacterial bioassay. The structural similarity search was used against Pubchem database through measurement of similarity with threshold between 70%-80% that it is increase possibility of selecting hits with same bioactivity (19). Lipinski rule of five and mycobacterial bioassay were then used to drug likeness and novelty. 

Finally, compounds were selected from virtual library hits for antimycobacterial bioassay. All of these compounds were commercially available chemical intermediates.

*Antimycobacterial bioassay*

The minimal inhibitory concentration of each compound against Bacillus Calmette–Guérin (BCG) was determined by the microplate alamar blue assay (20). Ethambutol was used as positive control drug. It was solubilized in DMSO to obtain drug stoke at 100 µg/mL concentration. Microbial suspension of BCG (1173P2) was diluted 1:10 with deionized water to reach to 0.5 Mcfarland. Selected chemical intermediates' solutions were prepared in DMSO at concentration of 1000 µg/mL. They were then serially diluted in 7H9 broth to reach 500, 250, 125, 62.5, 31.25, 15.62, 7.8 and 3.9 µg/mL in microplates (n=2). The microbial suspension of BCG was added to compounds dilutions and incubated at 37 °C for 4 days, and then 20 μL of 0.01% alamar blue solution with 12 μL of 10% Tween 80 was added to each well. The color change from blue to pink was observed after 48 h, and 72 h reincubation at 37 °C, and the minimal inhibitory concentration (MIC) was defined as the lowest concentration of compounds that inhibited a color change (21).

*Cell cytotoxicity assay and selectivity index (SI)*

The Human Umbilical Vein Endothelial (HUVEC) cell line (NCBI Code: C554) was used to assess cell cytotoxicity. The cells were centrifuged at 3000 rpm for 5 min. Cell pellets were resuspended in complete culture media at the concentration of 100,000 cells per mL. 100 µL of untreated cells were added to each well and incubated for 24 h. 100 µL of each compounds were then added to each well to reach final concentration of 1000, 500, 250, 125, 62.5 and 31.25 µg/mL in each wells (n=3). DMSO and doxorubicin (4 µM) were solvent control and positive control, respectively. Plates were then incubated for 24 h at 37 ˚C, with 5% CO_2_ in a humidified incubator. Cell viability was assessed using MTT tetrazolium dye according to Mosmann *et al.* (22) and Denizot and Lang (23). 11 µL of MTT tetrazolium dye (5 mg/mL) were added to each well and incubated in 37 ˚C for 5 h. The insoluble formazan formation was dissolved in 100 µ1 DMSO. Optical density (OD) was recorded using an ELISA reader (Organon Tekninka, The Netherlands) at wavelengths of 570 and 630 nm. % Viabilty and %Cytotoxicity were calculated by Equation 1 and 2.

Equation 1 $$\mathrm{\%Viability}=\left(\frac{\mathrm{mean absorbance of treated cells}}{\mathrm{mean absorbance of negative control}}\right)\times 100$$

Equation 2 %Cytotoxicity = 100 - %Viability

The ratio between IC_50_ and MIC against BCG was used to calculate the selectivity index of each compound for identification of potent hits. Favorable profile was assessed by selectivity index (SI) more than 10 µg/mL which was suggested safe for further screening (24, 25).

## Results

*Selected drug target candidates*

This step involved integration of six datasets from different selection criteria. Selection of *Mycobacterium *targets was then performed via integrated criteria. As shown in Table 1, three targets of metabolic pathways, pantothenate synthetase (*PanC*), anthranilate phosphoribosyl transferase (*TrpD*) and 3-isopropylmalate dehydratase, small subunit (*LeuD*), contained all of these selection criteria. These results suggest that these three targets might be promising drug target candidates (Table 1).

**Table 1:** Selected drug target candidates via integrated criteria in *Mycobacterium*

**Selection Criteria**	**Role of Criteria **	***panC*** a	***trpD*** b	***leuD*** c	**Dataset **
Known function	Rational drug design and probable study	+	+	+	Bioinformatic tools: 1) KEGG (7) 2) TubercuList (8)
Essentiality	Vital for *Mycobacterium *growth and survival	+	+	+	Bioinformatic tool: TubercuList (8)
Choke points	Biochemical lethality of metabolic networks	+	+	+	Kushwaha and Shakya *et al.,* 2010 (11)
Non-homology	Prevent of side effects	+	+	+	Anishetty *et al.* 2005 (12)
Involvement in persistence	Important for persistent or latent bacilli	+	+	+	Bioinformatic tool: TB Database (14)
Involvement in virulence	Important for pathogenesis	+	+	+	Bioinformatic tool: VFDB (16)

a Pantothenate synthetase

b Anthranilate phosphoribosyl transferase

c 3-isopropylmalate dehydratase, small subunit

*Selected ligand*

**Figure 1:**
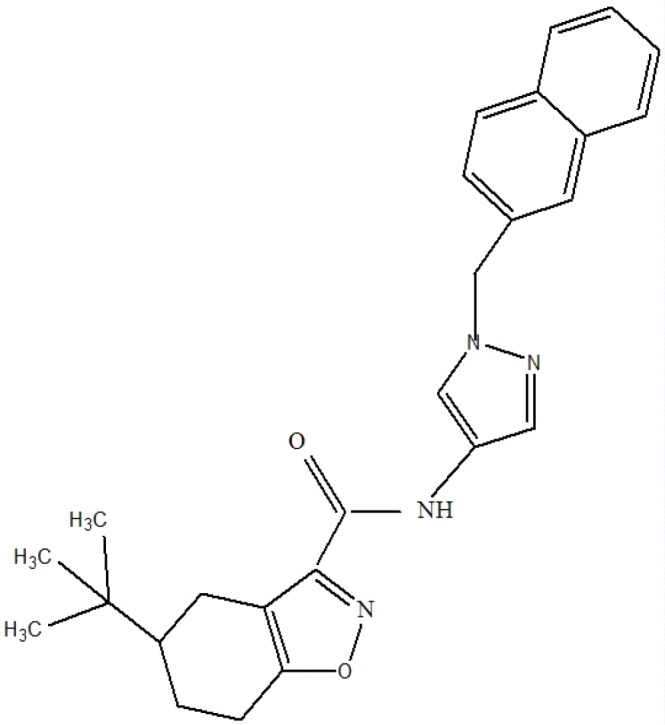
The selected primary ligand used for virtual screening, which is also complied with Lipinski rule of five.

Result showed that pantothenate synthetase is the only target candidate that had tractable ligands. Ligand properties were evaluated to select the best for antimycobacterial design. In this step, Lipinski rule of five and enzyme activity were used for further consideration of drug-likeness and ligand potency, respectively. Most of these ligands followed Lipinski rule of five. But they acted differently in enzyme activity ranging from nm to µM. The best ligand which had IC_50_ value of 90 nM and obeyed Lipinski rules of five, was selected for virtual screening (Figure 1).

*Ligand based virtual hits*

In the first step, 57916 hits were obtained through ligand based similarity search. These similar hits were filtered through Lipinski rule of five to obtain 2239 hits. The obtianed similar drug like hits were then filtered through applying mycobacterial bioactivity. About 650 compounds with mycobacterial bioactivity were eliminated through this filtration step. Remaining 1589 compounds were then investigated regarding whether they are commercially available chemical intermediates or not. At the end, 12 chemical intermediates were selected for subsequent antimycobacterial bioassay. These chemical intermediates and some of their Lipinski related physicochemical properties are shown in Table 2. It should be noted that the screened inhibitors were synthesized from chemical intermediated. The synthetic derivatives were then confirmed by H-NMR (not shown in this article).

**Table 2:** Selected compounds of virtual library compounds and their Lipinski related physicochemical properties.

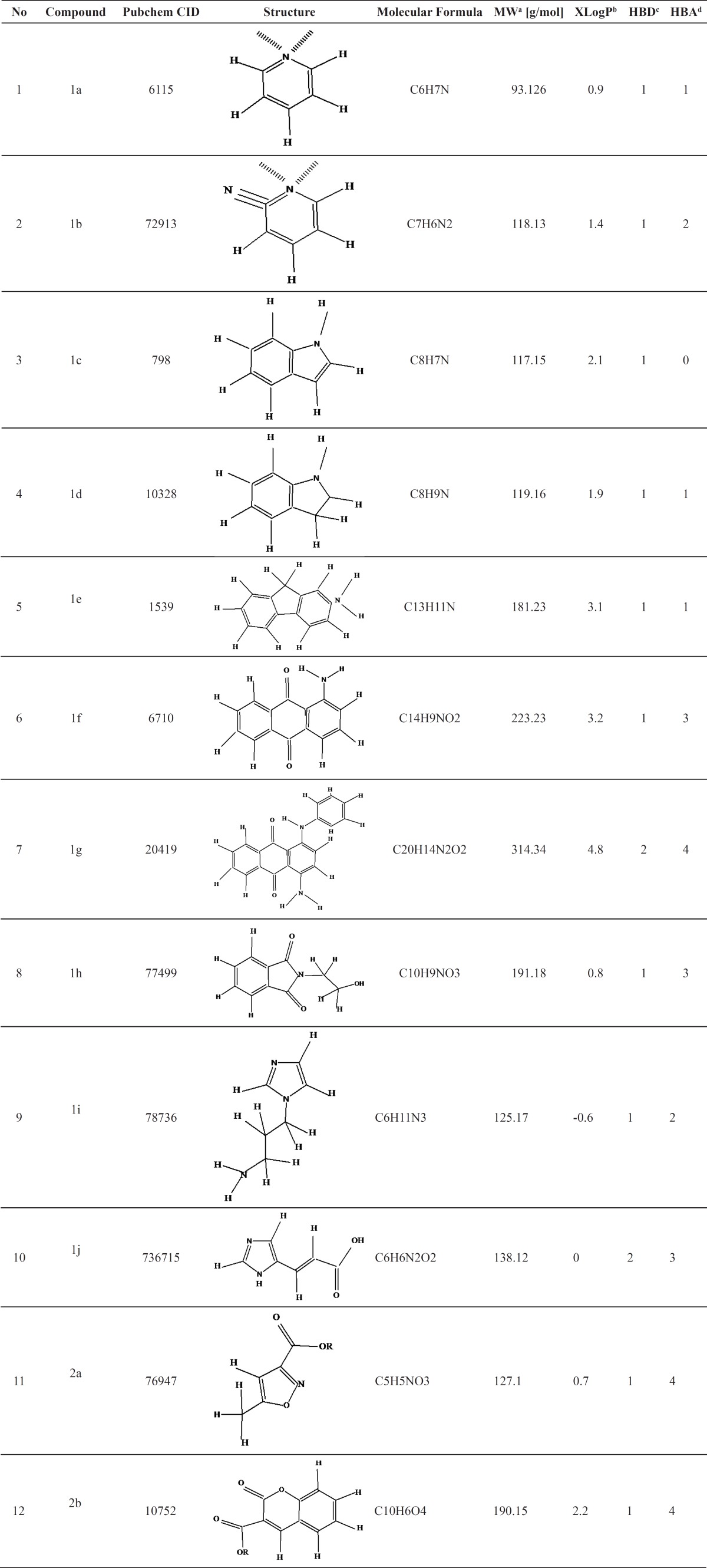

^a^ Molecular Weight

^b ^Primary predictive index of lipophilicity (Log Octanol/Water Partition), (XlogP)

^c^ H-Bond Donor

^d^ H-Bond Acceptor

*Antimycobacterial bioassay*

The chemical intermediates and their antimycobacterial activities are listed in Table 3. Six chemical intermediates showed inhibition of *Mycobacterium* bovis with minimum inhibitory concentration (MIC) of <46.8 µg/mL. The most active compounds were 1g, 1d, 1e and 1f with MICs of <3.9, 7.81, 11.7175, 7.8 μg/mL, respectively. Compounds 1c and 2b had acceptable activities (MIC = 46.875 and 31.25 μg/mL) among the other chemical intermediates.

All in all, *panc* primary ligands lacked favourable whole bacterium bioassay. But, virtual hits were shown improved antimycobacterial activity in comparison to *panc* primary ligands. Antimycobacterial bioassay results are shown in Table 3.

**Table 3:** Antimycobacterial activity data of the tested compounds.

**No**	**Compound**	**MIC (µg/mL)**	
		**48 h**	**72 h**
1	1a	500	250
2	1b	500	93.75
3	1c	125	46.875
4	1d	31.25	7.81
5	1e	31.25	11.7175
6	1f	187.5	7.81
7	1g	7.81	<3.9
8	1h	500	500
9	1i	250	125
10	1j	500	500
11	2a	500	125
12	2b	125	31.25

MIC of ethambutol: 3.125 μg/mL.

*Cell cytotoxicity and selectivity index (SI)*

Cytotoxicity determination of screened compounds is crucial in prediction of their fate in lead compounds development in drug discovery process. The cytotoxicity assay of all chemical intermediates against HUVEC cell line was tested. The results were used to calculated selectivity index. The majority of these compounds (11 out of 12), had IC_50_ value of more than 100 µg/mL which has acceptable cytotoxicity range. 1-aminoanthraquinone was the only toxic compound with IC_50 _ of 14.17 µg/mL and so was omitted in this step. Non- toxic effects of the proposed compounds are of great importance. Because cytotoxicity disapproval has always led to discontinue further studies on drug candidates and drug delivery systems (26).

The ratio of IC_50_ and MIC against BCG was used to calculate the selectivity index of each compound. The selectivity index (SI) equals and more than 10, was chosen for antimycobacterial screening procedure. The screening ended in five chemical intermediates with good selectivity index. These compounds were shown to inhibit BCG growth with low mammalian cell toxicity (Table 4). 

**Table 4:** Cell cytotoxicity and selectivity index data

No	**Compound**	**BCG MIC** **µg/mL**	**MTT IC**_50_ **µg/mL**	**SI (IC**_50_ **/MIC)** **µg/mL**
1	1a	250	1037.522	4.150088
2	1b	93.75	907.784	9.683029
3	1c	46.875	573.15	12.2272
4	1d	7.81	800.164	102.4538
5	1e	11.7175	726.325	61.98635
6	1f	7.81	14.17	1.814341
7	1g	<3.9	344.481	>88.32846
8	1h	500	841.506	1.683012
9	1i	125	440.508	3.524064
10	1j	500	1186.853	2.373706
11	2a	125	1092.841	8.742728
12	2b	31.25	924.271	29.57667

IC_50 _of doxorubicin: 3.1 μg/mL.

## Discussion

The global pipeline for new anituberculars is not sufficient to meet widespread concerns about this infectious disease (1-3). High burden of tuberculosis especially in developing countries where are high risks for HIV has enforced investigators efforts and government funding to further improve pharmaceutical pipelines. 

Computer mediated techniques in drug discovery and development have become more popular in recent years. Computer-aided drug design (CADD), computational drug design, computer-aided molecular design (CAMD), computer aided molecular modeling (CAMM), rational drug design, *in-silico* drug design, computer-aided rational drug design are common terms in this approach of drug discovery (27).

Computer-aided drug design is being utilized to find out hits or active drug candidates, to select lead compound applied for further evaluations and to optimize their physiochemical and pharmacokinetic properties in biological systems. Another computer mediated method is virtual screening which is to discover new drug candidates by searching chemical structure databases to find appropriate chemical hits (11, 12, 28, 29). 

The goal of these methods is to enrich the number of molecules with desirable drug like while eliminating undesirable toxic properties and serious adverse events. In another words, *in-silico* approach is a method of choice which significantly diminish time consuming and resource demanding for chemical synthesis and *in-vivo* testing of drug candidates (30, 31).

Literature review had showed that despite considerable efforts made in discovery and clinical evaluation of new lead compounds, most of promising validated targets for tuberculosis failed to meet required outcomes (6, 24). Reviewing establish guidline for tuberculosis also revealed that, only one new antitubercular drug, named Sirturo, has been approved during last 40 years (4, 5). Therefore it can be said that, availability of new drug targets has not been the limiting factor by now. The more important point is the possibility of chemoinformatics follow up. We have used *in-silico* ligand analysis in this study to increase chemical tractability of targets that are more likely to proceed into chemoinformatics follow up. 

Pantothenate synthetase (PS; EC 6.3.2.1) comprised a potential platform in *in-silico* target and ligand analysis. There is no approved drug that target pantothenate synthetase, so it would be an appropriate platform in drug design. That simply explains why we chose this target in cheminformatics follow up for the development of new antimycobacterial agents.

Pantothenate synthetase is known to catalyze an essential step in the de novo biosynthesis of pantothenate through a Bi Uni Uni Bi Ping Pong kinetic mechanism (32). The PS reaction proceeds through two steps. The first part of reaction is ATP dependent formation of a pantoyl adenylate intermediate. In the second part, nucleophilic attack of β-alanine on the activated carbonyl group of intermediate lead to formation of AMP and pantothenate (33).

Several approaches have been applied in design for pantothenate synthetase inhibitors. These included high-throughput screening (HTS) (34-36), analogues design through mimicking intermediate reactions (33), and fragment-based approach (37, 38).

High throughput screening system has developed to screen a small library of compounds for inhibitor design. Inhibitor examples were resulted from this approach is nafronyl oxalate and actinomycin D. They had poor activity against pantothenate synthetase. Some strategies like molecular mechanism determination would be helpful in finding effective inhibitors from HTS model (34, 36).

Another research based study on HTS model has performed by Velaparthi *et al.* (35). They have made further modifications in the active compounds of HTS model through docking insights in modeling process. Inhibitory effect of these compounds were found to be IC_50_ in range of 90 nM to 7.13 μM for PS and Minimum inhibitory concentration of Mtb > 128 μM. At high concentration of PS, this inhibitory effect could be affected by off-target toxicity of PS and its metabolites. They proposed that a drastic modification of the main scaffold would be required to reduce pharmacokinetic and adverse drug effects (35). 

Active ligand selected for further virtual screening obtained from the referred study. They reported better IC_50_ for PS in comparison to other studies. Ligand-based virtual screening was then performed to improve HTS model hits. Finally, five out of twenty one selected virtual library hits showed good activity in antimycobacterial bioassay. These compounds had good inhibitory activity in comparison to primary compounds in Velaparthi’s study. This result may prove the hypothesis that ligand based virtual screening strategy based on filtration is an improving strategy to find effective inhibitors from HTS model. These potent inhibitors with less toxicity are expected to be useful in designing inhibitors of *Mycobacterium* through structure based drug design in future studies.

Ligand-based virtual screening approach was used to identify the intermediate compound with appropriate antimycobacterial activity. These compounds possibly providing the seeds for novel antimycobacterial leads. There was a gap in previous computational methods such as virtual screening. Limited use of library input filtration through Lipinski rules was the main reason for limited number of hits with drug likeness or lead likeness in antitubercular drug discovery. Early consideration of such factors has been advocated to improve ultimate success in drug identification (30, 31). Therefore, Lipinski rules of five were used in first steps of this study. 

In general, experimental exploration of target in the whole cell is more acceptable than single target. It is simply because potent enzyme inhibitors frequently fail to translate into agents that will kill or even inhibit growth. It worth saying that, many successful antibacterial have multiple targets. Some of the key antimycobacterials such as isoniazid, required after uptake metabolic activation to exert their bactericidal effects (39) Therefore, whole cell bioassay against BCG procedure was used as the efficacy testing in this study.

At the end, five chemical intermediates include indole (1c), indoline (1d), 2-fluorenamine (1e), 1-amino-4-anilino-9,10-anthraquinone (1g) and coumarin-3-carboxylic acid (2b) showed inhibition of *Mycobacterium* *bovis* with good selectivity indices (SI) ≥10 (25).

Indole (1c) and indoline (1d) had good activity against *Mycobacterium* with MIC values of 46.875 and 7.81 µg/mL, respectively. Indole and indoline activity against *Mycobacterium* have also been in agreement with other researchers (40-42). The most important result of previous study has been the tuberculostatic effect of indole was comparable with Isoniazid (INH) (40). In this study, antimycobacterial activity of indoline is more than indole. Previous research has focused on anti-TB activity of Schiff base derivatives of indolin-2,3-dione (isatin) (42) but it seems that more investigation on indoline derivatives is needed.

The compound 5-methoxyindole has previously been identified from fragment-based screen against pantothenate synthetase of *Mycobacterium tuberculosis*. The precise binding mode has revealed through crystal structure analysis of 5-methoxyindolen which bound to PS. It revealed two key hydrogen-bonding interactions. One takes place between the OMe group of the indole and the backbone nitrogen atom of Val187. The other H bond occurs between the indole NH group and sulfate, which itself interacts with the Ser197 backbone nitrogen atom and the Lys160 residue (37). We propose similar mechanism for derivatives of indole and indoline. 

In this study, two of three anthraquinone based compounds (1f and 1g) were found to exhibit high antimycobactrial activity with MIC values of 7.81 and <3.9 µg/mL. But cytotoxicity of 1f in HUVEC cell line was not in acceptable range. It can be said that some anthraquinone containing compounds might act as novel potential therapeutics in the future. 

Recently, molecular hybridization of the phthalimide subunit that present in thalidomide and sulphonamide drugs has developed antimycobacterial compounds against *M. leprea* and *M. tuberculosis *(43)*.* This hypothesis was tested in compound 1 h. Unfortunately, N-(2-Hydroxyethyl)-phthalimide (1h) did not show good activity but additional studies on the antimycobacterial activity of N- phthalimide derivatives are currently underway in our laboratory.

Certain imidazole based compounds such as nitroimidazole series, PA-8242 and OPC-676833, which has showed good *in-vitro* and *in-vivo* activity against *mychobacterium tuberculosis* in both active and persistent form have entered into clinical studies and they are being evaluated. Since then nitroimidazoles explored as a promising scaffold for the antituberculars development (44-47). Unfortunately, our study showed 1j (imidazole based compound) had no activity against *Mycobacterium*.

The compound 5-methylisoxazole-3-carboxylic acid had no antimycobacterial activity in this study. Several reports in the literature has demonstrated the antitubercular activity of isoxazole scaffold. Some of these compounds were exhibited nanomolar activity against the replicating bacteria (R-TB) and low micromolar activity against the non-replicating bacteria (NRP-TB) (48). 

On the other hand, coumarin-3-carboxylic acid (2b) generated good activity and exhibited MIC values of 31.25. Several reports has demonstrated antitubercular activity of coumarin scaffold (49, 50). A series of coumarins with mono- and disubstituted benzaldehydes substitution in position 3 was studied against *M. tuberculosis*. These compounds were exhibited a significant activity (50–100 µg⁄mL) when compared with the first-line drugs (50). 

Amongst 12 chemical intermediates, some of them exhibited a pronounced activity against *M. bovis BCG* with good selectivity index values. These derivatives can be considered as starting points for further modifications to reach compounds with promising antitubercular activity to enter into other complicated assays involving mechanism of action, *in-vivo* tests and perhaps clinical trials.

## Conclusion

In the present study, we seek target and ligand *in-silico* techniques with the aim of avoiding the time-consuming and costly step. Both Lipinski rule of five and bioassay against *Mycobacterium* were used to optimize the efficiency of virtual screening and resulted in identifying more potent and efficient antimycobacterial compounds. After biological assay, we identified five antimycobacterials with selectivity index of ≥ 10. Therefore we suggest that this compound might be helpful to identify new hits with better activity profile against Mycobacterium and are proposed for further studies.
